# Enhanced Systemic Antitumour Immunity by Hypofractionated Radiotherapy and Anti-PD-L1 Therapy in Dogs with Pulmonary Metastatic Oral Malignant Melanoma

**DOI:** 10.3390/cancers15113013

**Published:** 2023-06-01

**Authors:** Tatsuya Deguchi, Naoya Maekawa, Satoru Konnai, Ryo Owaki, Kenji Hosoya, Keitaro Morishita, Motoji Nakamura, Tomohiro Okagawa, Hiroto Takeuchi, Sangho Kim, Ryohei Kinoshita, Yurika Tachibana, Madoka Yokokawa, Satoshi Takagi, Yukinari Kato, Yasuhiko Suzuki, Shiro Murata, Kazuhiko Ohashi

**Affiliations:** 1 Veterinary Teaching Hospital, Faculty of Veterinary Medicine, Hokkaido University, Sapporo 060-0819, Japan; 2 Department of Advanced Pharmaceutics, Faculty of Veterinary Medicine, Hokkaido University, Sapporo 060-0818, Japan; 3 Department of Disease Control, Faculty of Veterinary Medicine, Hokkaido University, Sapporo 060-0818, Japan; 4 Department of Veterinary Surgery 1, School of Veterinary Medicine, Azabu University, Sagamihara 252-5201, Japan; 5 Department of Antibody Drug Development, Tohoku University Graduate School of Medicine, Sendai 980-8575, Japan; 6 Department of Molecular Pharmacology, Tohoku University Graduate School of Medicine, Sendai 980-8575, Japan; 7 International Institute for Zoonosis Control, Hokkaido University, Sapporo 001-0020, Japan; 8 Global Station for Zoonosis Control, Global Institution for Collaborative Research and Education (GI-CoRE), Hokkaido University, Sapporo 060-0808, Japan

**Keywords:** hypofractionated radiation therapy, anti-PD-L1 antibody, immune checkpoint blockade, canine malignant melanoma, immunotherapy

## Abstract

**Simple Summary:**

Immune checkpoint inhibitors (ICIs) are promising treatment options for spontaneous cancers in dogs. To optimize the clinical benefit of ICIs, combination therapy with radiation has been proposed for human cancers; however, the safety and efficacy of the combination approach have not yet been reported in dogs. The retrospective analysis of dogs bearing pulmonary metastatic oral malignant melanoma suggested that the use of previous (≤8 weeks) hypofractionated radiation therapy is associated with a better clinical response to anti-PD-L1 immunotherapy. Radiotherapy before the initiation of anti-PD-L1 therapy can be an effective approach to enhance antitumor immunity in dogs.

**Abstract:**

Although immune checkpoint inhibitors (ICIs), such as the anti-programmed death-ligand 1 (PD-L1) antibody, have been developed for the treatment of canine malignant melanoma, desirable clinical efficacies have not been achieved. Recent studies in humans have suggested that radiation therapy (RT) combined with ICIs induces robust systemic antitumour immunity in patients with cancer. This study retrospectively examined the therapeutic efficacy of combination therapy (hypofractionated RT and anti-PD-L1 antibody [c4G12]) in dogs with pulmonary metastatic oral malignant melanoma. The intrathoracic clinical benefit rate (CBR)/median overall survival (OS) in the no RT (*n* = 20, free from the effect of RT), previous RT (*n* = 9, received RT ≤8 weeks prior to the first c4G12 dose), and concurrent RT (*n* = 10, c4G12 therapy within ±1 week of the first RT fraction) groups were 10%/185 days, 55.6%/283.5 days (*p* < 0.05 vs. no RT group), and 20%/129 days (*p* > 0.05 vs. no RT group), respectively. The adverse events were considered to be tolerable in the combination therapy. Thus, hypofractionated RT before the initiation of c4G12 therapy can be an effective approach for enhancing the therapeutic efficacy of immunotherapy, with acceptable safety profiles. Further prospective clinical studies are required to confirm the findings of this study.

## 1. Introduction

Melanoma is the most common oral malignancy observed in dogs. The biological behaviour of canine oral melanoma has been considered locally invasive and highly metastatic [1,2] and is comparable to that of human oral melanoma [3]. Radiation therapy (RT) is an effective treatment for primary oral melanoma and involved lymph nodes. Hypofractionated protocols (e.g., 3–6 once-weekly fractions of 6–9 Gy) have often been used to treat canine oral malignant melanoma (OMM) [4,5,6]; however, the optimal dose per fraction, fraction numbers, and intervals have yet to be determined. The reported range of overall response rate of RT was 73–94%, whereas the progression-free interval ranged from 3.6–8.6 months [7,8,9,10,11]. The high metastatic potential, especially to the lung, makes it difficult to achieve long-term control of the tumour and ultimately leads to the death of the affected dogs [4,12]. The establishment of systemic therapy is necessary to improve the prognosis of canine OMM because conventional chemotherapy does not improve the survival time [13,14]. Although several immunotherapies have been proposed for the treatment of canine OMM, desirable clinical efficacies have not been achieved [15].

RT has been associated with enhanced antitumour immunity not only locally but also systemically. Damage-associated molecular pattern molecules (DAMPs) are released when irradiation causes immunogenic cell death (ICD) in tumour tissues. Dendritic cells are stimulated by DAMPs through pattern recognition receptors, such as toll-like receptors, and activate cytotoxic T lymphocytes. Subsequently, activated T lymphocytes systemically attack tumour cells [16,17]. The enhancement of systemic antitumour immunity by irradiation has been recognised as an abscopal effect where metastatic lesions outside the irradiation field shrink after local RT. Apart from mouse cancer models, only 46 cases of confirmed abscopal effects have been reported in the literature from 1969 to 2014 [18]. Moreover, the induction of the abscopal effect in naturally occurring canine cancers has not been reported [19]. Thus, the enhancement of antitumour immunity by irradiation rarely occurs in spontaneous cancers in both humans and animals. The immune evasion mechanisms of tumours can counteract irradiation-induced antitumour immunity in the vast majority of cancer patients [20,21,22,23,24].

Programmed death 1 (PD-1) and its ligands, programmed death-ligand 1 (PD-L1) and PD-L2, are prominent molecules involved in tumour immune evasion and are called immune checkpoint molecules. These molecules play important roles in immune suppression to maintain immune homeostasis and protect the host from excessive immune responses or autoimmune diseases. PD-L1 and PD-L2 are type I transmembrane proteins that belong to the B7 family. Although the expression of PD-L2 is limited to dendritic cells, macrophages, and bone-marrow-derived mast cells, PD-L1 is expressed in various cell types, including haematopoietic and nonhematopoietic cells [25]. Overexpression of PD-L1 is a common feature of various human cancers, and it causes immune evasion of tumour cells by inhibiting cell proliferation and cytokine secretion of PD-1–expressing tumour-specific T cells [26,27]. Several types of canine malignant tumours also express PD-L1, including squamous cell carcinoma, nasal adenocarcinoma, transitional cell carcinoma, osteosarcoma, and malignant melanoma, suggesting that canine tumours have an immune evasion mechanism through PD-L1 overexpression [28,29,30,31,32,33,34,35,36]. In our previous reports, almost all OMM cases tested expressed PD-L1 (19/20 cases, 95%) [35]. Recent studies have demonstrated that treatment with canine chimeric anti-PD-L1 antibody (c4G12) is effective against canine OMM [33,35]. c4G12 binds to canine PD-L1 with high avidity, efficiently inhibits the interaction between canine PD-1 and PD-L1, and enhances cytokine production and T-cell proliferation in canine peripheral blood mononuclear cell cultures [33]. The response rate to c4G12 treatment in canine OMM was found to be 14.3% (1/7 dogs) [33], which is comparable to that of human anti-PD-L1 antibody therapy in patients with advanced melanoma [37]. Although anti-PD-L1 therapy has achieved a breakthrough for this extremely progressive tumour, further analysis is needed to improve the response rate and survival benefit.

In irradiation-induced antitumour immunity, the PD-1/PD-L1 axis may play a crucial role in the suppression of cytotoxic T cell responses within the tumour microenvironment because PD-L1 expression is upregulated after irradiation [38]. Therefore, the combination of RT and anti-PD-1/PD-L1 therapy has gained attention for achieving synergistic antitumour efficacy. Indeed, the combination therapy of RT and immune checkpoint inhibitors (ICIs) induced an abscopal effect in mouse models of cancer [24,39], and early clinical studies have demonstrated that patients with non-small-cell lung cancer who received previous RT had an improvement in overall survival (OS) after anti-PD-1 antibody treatment as compared to those without previous RT [40].

In this study, we evaluated the synergistic antitumour efficacy of hypofractionated RT and c4G12 treatment in dogs with pulmonary metastatic OMM. To investigate whether the combination therapy induced better systemic antitumour immunity, dogs treated with c4G12 at the Hokkaido University Veterinary Teaching Hospital (HUVTH) were retrospectively evaluated for the response rate of intrathoracic (i.e., outside the irradiation field) metastatic lesions and OS. Dogs were divided into three groups based on the presence or absence of RT, as well as the timing of RT, to examine whether the temporal order of the combinational irradiation influences the outcome of c4G12 immunotherapy.

## 2. Materials and Methods

### 2.1. Entry Requirement

Dogs with pulmonary metastatic OMM (stage IV in the TNM-based staging scheme) that underwent c4G12 treatment at HUVTH between March 2016 and September 2021 were retrospectively examined in this study. All dogs were diagnosed with OMM based on histopathological examination and PD-L1 expression was assessed by immunohistochemical staining as described previously [35]. Pulmonary metastatic sites were detected using chest radiography or computed tomography (CT) in all dogs. Dogs with concurrent cancer of different origins or severe systemic illness unrelated to the cancer were excluded from the analysis. The clinical study using c4G12 was conducted with the approval of the Institutional Animal Care Committee of Hokkaido University (Approval number: 15-0149 and 20-0041). The use of animals throughout the clinical study was approved by the Ethics Committee of the Faculty of Veterinary Medicine, Hokkaido University. Prior to enrolment in the clinical study, written informed consent was obtained from the dog owners.

### 2.2. c4G12 Treatment

c4G12 treatment was performed as described previously [35]. Dogs received intravenous administration of c4G12 every 2 weeks at 5 mg/kg (in some cases, 2 mg/kg was chosen by veterinarians) over 1 h. Results of physical examination, complete blood count, and blood chemistry were used to evaluate adverse events. Adverse events were graded and recorded according to the Veterinary Cooperative Oncology Group–Common Terminology Criteria for Adverse Events (VCOG-CTCAE) v1.1 [41].

### 2.3. Radiation Therapy

Most RT treatments were conducted using the Elekta Synergy Integrity R LINAC (Elekta AB, Stockholm, Sweden), whereas some dogs were treated using an orthovoltage machine, TITAN-320S (Shimadzu Industrial System, Kyoto, Japan). In all dogs, general anaesthesia was induced with propofol and maintained with sevoflurane delivered in oxygen. The oral tumour and involved lymph nodes, if present, received four fractions of RT (approximately 26–32 Gy in total) at one-week intervals. For the linac radiation treatment, the gross tumour volume (GTV), clinical target volume (CTV), planning target volume (PTV), and organ at risk (OAR), including the eyes, brain, and larynx, were delineated using the CT dataset. Inverse planning was designed with isocentrically placed 4 or 6 MV radiation beams using volumetric modulated arc therapy (VMAT) techniques with one arc on the CMS Monaco 3.0 treatment planning system (CMS-Elekta Ltd., Crawley, UK). It utilises a Monte Carlo algorithm with continuous dose rate variation to determine the dose with heterogeneity correction. Forward planning was performed with 4, 6, and 10 MV beams using 3-dimentional conventional RT on a Pinnacle 3 treatment planning system (Philips Healthcare, Best, The Netherlands). Orthovoltage treatment planning was designed based on hand calculation for a 200 kV photon beam. A few dogs (*n* = 3) received RT in other facilities before their first visit to HUVTH, with unspecified protocols and instruments.

### 2.4. Evaluation of Intrathoracic Response

Tumour response in the thoracic cavity (i.e., pulmonary metastatic lesions) to c4G12 treatment was recorded according to the response evaluation criteria for solid tumours in dogs (cRECIST) v1.0 [42]. Baseline assessments were performed within three weeks prior to the first c4G12 dose. During treatment, tumours were evaluated at least every 6 weeks using the same modality (chest X-ray or CT). For dogs with non-measurable disease (i.e., all metastatic lesions in the lung <10 mm on CT or <20 mm on X-ray), tumour response was classified as either CR (disappearance of all detectable tumours), PD (unequivocal progression of the tumour or appearance of new lesions), or SD/PR (reduction or stable persistence of the tumour: not classified as CR or PD). The CBR was defined as the percentage of dogs with the best intrathoracic response of CR, PR, SD, or SD/PR.

### 2.5. Evaluation of Survival

OS (days) of dogs was defined as the time from the first c4G12 dose to death. In comparison with historical data of dogs treated with RT only, OS was defined as the time from the first dose of RT to death. Dogs with pulmonary metastatic OMM treated with RT only at HUVTH between 2014 and 2019 were included in the historical control group (*n* = 10, RT-only group). Kaplan–Meier curves were generated to estimate the median survival time with a 95% CI. Dogs that were still alive at the end of the study period were included in the survival analysis as censored data.

### 2.6. Statistical Analysis

Statistical analyses of binary variables, continuous variables, and survival times were performed using Fisher’s exact test, the Kruskal–Wallis test followed by the Steel-Dwass test, and the log-rank test, respectively. The statistical software EZR (version 1.35) [43] was used for all the analyses. *p* < 0.05 was considered statistically significant.

## 3. Results

### 3.1. Baseline Characteristics of Dogs

**Figure 1:**
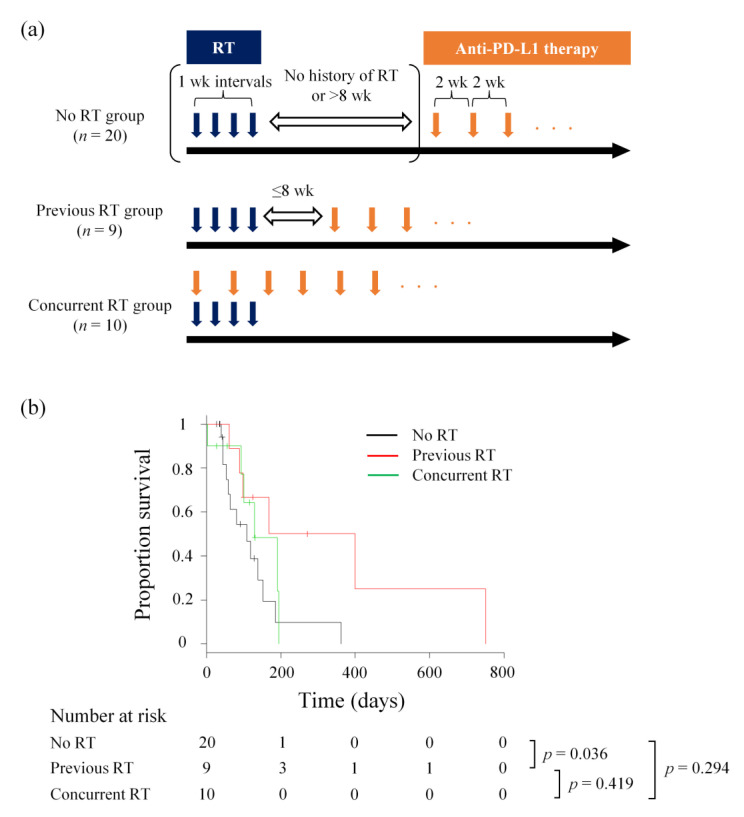
OS of dogs receiving c4G12 immunotherapy with or without previous/concurrent RT. (**a**) Timing of RT and anti-PD-L1 antibody (c4G12) therapy for each treatment group. (**b**) Kaplan–Meier curves for OS from the initiation of c4G12 immunotherapy. Marks on the line indicate censored data. Statistical analysis was performed using the log-rank test.

In total, 39 dogs with pulmonary metastatic OMM were eligible, including the 26 dogs described in a previous report [35], and included 12 miniature dachshunds, five mixed-breed dogs, three pugs, three golden retrievers, three toy poodles, two American cocker spaniels, two Chihuahuas, two Labrador retrievers, and one each of the following: beagle, Yorkshire terrier, kaninchen dachshund, papillon, flat-coated retriever, Pomeranian, and Pekingese. The median age was 13 years (range, 8–15 years), with 21 males (15 castrated) and 18 females (13 spayed). The median body weight was 6.0 kg (range, 2.0–29.0 kg). The primary tumour locations were mandible (*n* = 20), maxilla (*n* = 17), and unspecified (*n* = 2) (Table S1). Dogs treated with c4G12 were separated into the following three groups: (1) no RT group, dogs that were considered free from the effect of RT, with no history of RT or the previous RT terminated >8 weeks before the initiation of c4G12 therapy, (2) previous RT group, dogs that were treated with RT ≤8 weeks prior to the first c4G12 dose, and (3) concurrent RT group, which received RT along with the c4G12 therapy, with the first c4G12 dose administered within ±1 week of the first fraction of RT (Figure 1a). Among the 39 dogs, 20, nine, and ten belonged to the no RT, previous RT, and concurrent RT groups, respectively. No statistically significant differences were observed in sex, age, body weight, or primary tumour location among the treatment groups (Table 1).

### 3.2. Treatment and Adverse Events

All dogs received at least one dose of c4G12, administered every 2 weeks at 2 or 5 mg/kg. The median duration of c4G12 treatment was 91 days (range, 2–750 days) with a median number of c4G12 dosage of six times (range, 1–54 times) (Table S2). In the no RT group, adverse events of any grade related to c4G12 treatment occurred in 12 dogs (60%). Most events were classified as grade 1–2; however, grade 3 events (pneumonitis and anaphylaxis) occurred in two dogs (10%). Both grade 3 events were resolved after short-term supportive care. In the previous RT group, adverse events of any grade were observed in four dogs (44%), including grade 3 events (elevation in alanine aminotransferase [ALT], aspartate aminotransferase [AST], or lipase) in three dogs (33%). In the concurrent RT group, adverse events of any grade occurred in six dogs (60%), including grade 3 events (elevation in alkaline phosphatase/AST or blood urea nitrogen [BUN]) in two dogs (20%). None of the grade 3 events in the previous and concurrent RT groups were associated with clinical symptoms; thus, additional care was not indicated. The frequency of grade 3 adverse events did not differ significantly among the groups (Table 2).

All dogs except for one in the concurrent RT group completed the RT protocol as planned (four fractions of 6.5–8 Gy at one-week intervals). Adverse events related to RT were typically grades 1–2 and were characterised by alopecia, pruritus, skin ulceration, and mucositis, which were resolved without treatment. To examine whether combination therapy increases the risk of side effects associated with RT, the frequency of adverse events related to RT was compared with the RT-only group (*n* = 10, historical control which consisted of dogs with pulmonary metastatic OMM treated with RT only, Table S3). No significant differences were observed in the frequency of adverse events related to RT among the RT-only, previous RT, and concurrent RT groups (Table S4).

Surgical resection of the metastatic lesion was performed during c4G12 treatment in one dog in the no RT group (lymph node), two in the previous RT group (lung and eye), and one in the concurrent RT group (spleen). No additional treatment was performed in the remaining 35 dogs (Table S1).

### 3.3. Intrathoracic Response and Overall Survival

Intrathoracic metastatic lesions in two dogs responded to c4G12 treatment (one complete response [CR] and one stable disease [SD]) and the clinical benefit rate (CBR) was 10.0% (2/20) in the no RT group. The dog that achieved CR survived for 362 days, while another dog with SD survived for 152 days. In the previous RT group, four, one, and four dogs exhibited CR, SD/partial response (PR), and progressive disease (PD), respectively. The intrathoracic CBR was 55.6% (5/9), which was significantly higher than that of the no RT group (*p* = 0.016, Table 3). Among the dogs with a clinical benefit (*n* = 5), the median OS was 399 days (95% confidence interval [CI]: 98–NA days). In the concurrent RT group, two dogs experienced SD/PR and the CBR was 20.0% (2/10), which was not significantly different from that of the other groups (*p* = 0.584 vs. no RT group and *p* = 0.170 vs. previous RT group). The OS of the two SD/PR dogs was 131 and 194 days, respectively.

At the end of the study period, zero, one, and one dogs were still alive in the no RT, previous RT, and concurrent RT groups, respectively. Twelve dogs were censored because they were lost to follow-up. One dog that experienced CR in the previous RT group died of an unrelated cause (chronic kidney disease). Twenty dogs died of metastasis progression, while the remaining four died of local progression of OMM (Table S2). The median OS from the first c4G12 treatment to death in all 39 dogs was 129 days (95% CI: 93–185 days). The median OS in the no RT group was 185 days (95% CI: 53–152 days). OS was significantly longer in the previous RT group than that in the no RT group (*p* = 0.036), with a median OS of 283.5 days (95% CI: 61–NA days). The median OS of the concurrent RT group was 129 days (95% CI: 2–NA days), with no significant difference in OS as compared to the no RT group (*p* = 0.294, Figure 1b). However, OS from the first fraction of RT was significantly longer in the concurrent RT group than that of the RT-only group (median OS: 48 days, 95% CI: 14–NA days, *p* = 0.036, Figure S1).

## 4. Discussion

This study showed that in dogs with pulmonary metastatic OMM, the intrathoracic tumour response rate and OS were significantly improved in the previous RT group compared with that of the no RT group. These results strongly suggest that systemic antitumour immunity reinvigorated by anti-PD-L1 therapy was enhanced by ICD induced by RT. Furthermore, since concurrent RT did not improve the response rate and OS, the timing of RT is found to be of great importance for achieving synergistic efficacy. As the types, frequency, and severity of adverse events related to c4G12 or RT in combination therapy were similar to those of each treatment alone, hypofractionated RT before c4G12 therapy can be an effective combination therapy with acceptable toxicity to treat canine OMM.

The abscopal effect has been shown in many types of human malignant neoplasms, such as hepatocellular carcinoma, lymphoma, renal cell carcinoma, and melanoma. Although the reported cases were substantially limited, seven of 46 cases (15.2%) were recorded in patients with melanoma. Additionally, the lungs are the most common metastatic sites, exhibiting an abscopal effect [18]. Thus, dogs with pulmonary metastatic OMM, which we focused on in this study, are considered a suitable model to investigate the abscopal effects or enhancement of antitumour systemic immunity after irradiation. Although the abscopal effect itself rarely occurs and has been poorly studied, immunotherapy combined with RT may increase the frequency of cases with an abscopal effect. As ICIs such as the anti-PD-1/PD-L1/cytotoxic T lymphocyte antigen-4 (CTLA-4) antibody enhance systemic antitumour immunity and local immunosuppression caused by RT is considered to be partly due to the upregulation of immune checkpoints [38], the combination of ICIs and RT has been gaining attention for their expected synergistic efficacies.

One retrospective study in humans showed the outcomes of 21 patients with advanced melanoma treated with ipilimumab, a CTLA-4 inhibitor, followed by RT. An abscopal effect was observed in 11 patients (52%), nine of whom had PR (43%) and two had SD (10%) [44]. Additionally, RT during anti-PD-1 therapy achieved a 48% response rate (including CR, PR, and SD) for non-radiated lesions in advanced melanoma patients [45]. Although the timing of RT and ICI therapy was different, a CBR of 55.6% for non-irradiated lesions of canine OMM in the previous RT group in the present study was comparable or preferable to those in the combination therapy in human advanced melanoma. Additionally, the combination of ICIs and RT was associated with prolonged OS as compared to monotherapy in human cancer patients [40,46,47]. Consistent with these observations, in this study, the OS was significantly longer in the previous RT group than that in the no RT group. Interestingly, the OS in the concurrent RT group was similar to that of the no RT group; however, it was significantly longer than that of the historical RT-only group. These results suggest that c4G12 therapy is somewhat effective in all situations, but concurrent RT does not result in a synergistic effect with c4G12 treatment.

The optimal RT protocol (timing, dose, and fractionation) for combined therapy remains a topic of debate [48]. An early study using mouse models of cancer revealed that concurrent but not sequential (starting 7 days after the last dose of RT) anti-PD-L1 therapy was associated with improved survival, indicating that concurrent RT may induce better synergistic effects [49]. However, in human clinical studies, controversial results have been reported, suggesting that the optimal timing of RT depends on various clinical conditions, including cancer types, patient characteristics, and RT protocols. For example, in patients with metastatic melanoma, concurrent RT combined with anti-PD-1 antibody resulted in a similar OS as compared to sequential therapy (RT followed by anti-PD-1 therapy) [50]. Another study in patients with stage III non-small-cell lung cancer showed that sequential anti-PD-L1 therapy after chemoradiotherapy was associated with better OS than that of chemoradiotherapy alone [47,51]. More recently, using mouse models, administration of anti-PD-1 antibody before irradiation has been shown to increase irradiation-induced death of polyfunctional effector CD8^+^ T cells within the tumour microenvironment, leading to the loss of systemic antitumour immunity [52]. As the RT protocol used in this study was four fractions at one-week intervals and c4G12 was administered every 2 weeks, immune activation by the first two administrations of anti-PD-L1 antibody in the concurrent RT group may have been abrogated by the following fractions of RT, diminishing the synergistic antitumour efficacy. Therefore, we propose that the optimal timing of RT for canine OMM would be immediately before the initiation of anti-PD-L1 therapy, with the last dose of RT completed before the first dose of anti-PD-L1 antibody, although this hypothesis should be further examined in prospective clinical studies.

## 5. Conclusions

Combination therapy using hypofractionated RT and an anti-PD-L1 antibody may be a promising strategy to induce robust systemic antitumour immunity. This study provides the first evidence which suggests that a sequential combination (hypofractionated RT followed by c4G12 therapy) is better than a concurrent combination to elicit synergistic therapeutic efficacy against canine OMM. The findings may be extrapolated not only to other canine cancers but also human cancers where hypofractionated protocols are used for RT, as dogs are considered clinically relevant models of human cancer with various genetic backgrounds and spontaneous development of cancer in immunocompetent settings. Further studies are necessary to determine the optimal protocol for such combination therapy.

## Figures and Tables

**Table 1:** Characteristics of dogs included in this study.

	No RT (*n* = 20)	Previous RT (*n* = 9)	Concurrent RT (*n* = 10)
Breed			
American cocker spaniel	1 (5)	1 (11)	0
Beagle	1 (5)	0	0
Chihuahua	1 (5)	0	1 (10)
Flat-coated retriever	1 (5)	0	0
Golden retriever	1 (5)	2 (22)	0
Kaninchen dachshund	1 (5)	0	0
Labrador retriever	1 (5)	1 (11)	0
Miniature dachshund	6 (30)	2 (22)	4 (40)
Papillon	1 (5)	0	0
Pekingese	0	1 (11)	0
Pomeranian	0	1 (11)	0
Pug	2 (10)	0	1 (10)
Toy poodle	1 (5)	0	2 (20)
Yorkshire terrier	1 (5)	0	0
Mix	2 (10)	1 (11)	2 (20)
Sex			
Male	10 (50)	7 (78)	4 (40)
Castrated	6 (30)	5 (56)	4 (40)
Intact	4 (20)	2 (22)	0
Female	10 (50)	2 (22)	6 (60)
Spayed	8 (40)	1 (11)	4 (40)
Intact	2 (10)	1 (11)	2 (20)
Age			
≤10 years	2 (10)	2 (22)	1 (10)
>10 years	18 (90)	7 (78)	9 (90)
Body Weight			
≤5 kg	6 (30)	2 (22)	1 (10)
5–10 kg	10 (50)	3 (33)	9 (90)
>10 kg	4 (20)	4 (44)	0
Primary tumour site			
Mandible	10 (50)	2 (22)	5 (50)
Maxilla	8 (40)	7 (78)	5 (50)
Unspecified	2 (10)	0	0

No. of dogs (%) are shown.

**Table 2:** Adverse events related to c4G12 treatment.

	No RT (*n* = 20)		Previous RT (*n* = 9)		Concurrent RT (*n* = 10)	
	Any Grade	Grade 3	Any Grade	Grade 3	Any Grade	Grade 3
Any	12 (60)	2 (10)	4 (44)	3 (33)	6 (60)	2 (20)
Anorexia	0	0	1 (11)	0	0	0
Diarrhoea	2 (10)	0	1 (11)	0	2 (20)	0
Vomiting	3 (15)	0	2 (22)	0	2 (20)	0
Alkaline phosphatase	1 (5)	0	0	0	2 (20)	1 (10)
ALT	5 (25)	0	3 (33)	1 (11)	1 (10)	0
AST	0	0	3 (33)	1 (11)	1 (10)	1 (10)
BUN	0	0	0	0	2 (20)	1 (10)
Albumin, low	0	0	1 (11)	0	0	0
Glucose, low	0	0	1 (11)	0	0	0
Lipase	2 (10)	0	1 (11)	1 (11)	1 (10)	0
CPK	0	0	1 (11)	0	0	0
Conjunctivitis/ocular surface disease	1 (5)	0	0	0	0	0
Thrombocytopenia	2 (10)	0	0	0	0	0
Pancreatitis	0	0	0	0	1 (10)	0
Pneumonitis/pulmonary infiltrates	1 (5)	1 (5)	0	0	0	0
Allergic reaction	0	0	0	0	1 (10)	0
Anaphylaxis	1 (5)	1 (5)	0	0	0	0

No. of dogs with event (%) are shown. Adverse events considered to be related to c4G12 immunotherapy by the investigators are listed. ALT, alanine aminotransferase; AST, aspartate aminotransferase; BUN, blood urea nitrogen; CPK, creatine phosphokinase.

**Table 3:** Intrathoracic response to c4G12 treatment.

	No RT (*n* = 20)	Previous RT (*n* = 9)	Concurrent RT (*n* = 10)
CR	1 (5)	4 (44)	0
SD or SD/PR *	1 (5)	1 (11)	2 (20)
PD	15 (75)	4 (44)	6 (60)
NE	3 (15)	0	2 (20)
Clinical benefit **	2 (10)	5 (56) †	2 (20)

No. of dogs (%) are shown. * Non-CR/non-PD in dogs with non-measurable pulmonary lesions at baseline. ** Clinical benefit = CR + PR + SD (SD/PR). † *p* < 0.05 by Fisher’s exact test (vs. No RT group). CR, complete response; PR, partial response; SD, stable disease; PD, progressive disease; NE, not evaluable.

## Data Availability

All data generated or analysed during this study are included in this published article (and its Supplementary Information files).

## Supplementary Materials

The following supporting information can be downloaded at: https://www.mdpi.com/article/10.3390/cancers15113013/s1, Table S1. Baseline characteristics of c4G12-treated dogs; Table S2. Summary of c4G12 treatment; Table S3. Summary of dogs in RT-only group; Table S4. Adverse events related to RT; Figure S1. OS of dogs treated with RT only or c4G12 immunotherapy plus concurrent RT.

## References

[1] Bostock D.E. (1979). Prognosis after surgical excision of canine melanomas. Vet. Pathol..

[2] Millanta F., Fratini F., Corazza M., Castagnaro M., Zappulli V., Poli A. (2002). Proliferation activity in oral and cutaneous canine melanocytic tumours: Correlation with histological parameters, location, and clinical behaviour. Res. Vet. Sci..

[3] Vail D.M., MacEwen E.G. (2000). Spontaneously occurring tumors of companion animals as models for human cancer. Cancer Investig..

[4] Freeman K.P., Hahn K.A., Harris F.D., King G.K. (2003). Treatment of dogs with oral melanoma by hypofractionated radiation therapy and platinum-based chemotherapy (1987–1997). J. Vet. Intern. Med..

[5] Murphy S., Hayes A.M., Blackwood L., Maglennon G., Pattinson H., Sparkes A.H. (2005). Oral malignant melanoma—The effect of coarse fractionation radiotherapy alone or with adjuvant carboplatin therapy. Vet. Comp. Oncol..

[6] Dank G., Rassnick K.M., Sokolovsky Y., Garrett L.D., Post G.S., Kitchell B.E., Sellon R.K., Kleiter M., Northrup N., Segev G. (2014). Use of adjuvant carboplatin for treatment of dogs with oral malignant melanoma following surgical excision. Vet. Comp. Oncol..

[7] Cancedda S., Rohrer Bley C., Aresu L., Dacasto M., Leone V.F., Pizzoni S., Gracis M., Marconato L. (2016). Efficacy and side effects of radiation therapy in comparison with radiation therapy and temozolomide in the treatment of measurable canine malignant melanoma. Vet. Comp. Oncol..

[8] Bateman K.E., Catton P.A., Pennock P.W., Kruth S.A. (1994). 0-7-21 radiation therapy for the treatment of canine oral melanoma. J. Vet. Intern. Med..

[9] Blackwood L., Dobson J.M. (1996). Radiotherapy of oral malignant melanomas in dogs. J. Am. Vet. Med. Assoc..

[10] Kawabe M., Mori T., Ito Y., Murakami M., Sakai H., Yanai T., Maruo K. (2015). Outcomes of dogs undergoing radiotherapy for treatment of oral malignant melanoma: 111 cases (2006–2012). J. Am. Vet. Med. Assoc..

[11] Tollett M.A., Duda L., Brown D.C., Krick E.L. (2016). Palliative radiation therapy for solid tumors in dogs: 103 cases (2007–2011). J. Am. Vet. Med. Assoc..

[12] Proulx D.R., Ruslander D.M., Dodge R.K., Hauck M.L., Williams L.E., Horn B., Price G.S., Thrall D.E. (2003). A retrospective analysis of 140 dogs with oral melanoma treated with external beam radiation. Vet. Radiol. Ultrasound.

[13] Rassnick K.M., Ruslander D.M., Cotter S.M., Al-Sarraf R., Bruyette D.S., Gamblin R.M., Meleo K.A., Moore A.S. (2001). Use of carboplatin for treatment of dogs with malignant melanoma: 27 cases (1989–2000). J. Am. Vet. Med. Assoc..

[14] Boria P.A., Murry D.J., Bennett P.F., Glickman N.W., Snyder P.W., Merkel B.L., Schlittler D.L., Mutsaers A.J., Thomas R.M., Knapp D.W. (2004). Evaluation of cisplatin combined with piroxicam for the treatment of oral malignant melanoma and oral squamous cell carcinoma in dogs. J. Am. Vet. Med. Assoc..

[15] Almela R.M., Ansón A. (2019). A review of immunotherapeutic strategies in canine malignant melanoma. Vet. Sci..

[16] Palata O., Hradilova Podzimkova N., Nedvedova E., Umprecht A., Sadilkova L., Palova Jelinkova L., Spisek R., Adkins I. (2019). Radiotherapy in combination with cytokine treatment. Front. Oncol..

[17] Frey B., Rubner Y., Wunderlich R., Weiss E.M., Pockley A.G., Fietkau R., Gaipl U.S. (2012). Induction of abscopal anti-tumor immunity and immunogenic tumor cell death by ionizing irradiation—Implications for cancer therapies. Curr. Med. Chem..

[18] Abuodeh Y., Venkat P., Kim S. (2016). Systematic review of case reports on the abscopal effect. Curr. Probl. Cancer.

[19] Fan T.M., Selting K.A. (2018). Exploring the potential utility of pet dogs with cancer for studying radiation-induced immunogenic cell death strategies. Front. Oncol..

[20] Ahn G.O., Tseng D., Liao C.H., Dorie M.J., Czechowicz A., Brown J.M. (2010). Inhibition of Mac-1 (CD11b/CD18) enhances tumor response to radiation by reducing myeloid cell recruitment. Proc. Natl Acad. Sci. USA.

[21] Chiang C.S., Fu S.Y., Wang S.C., Yu C.F., Chen F.H., Lin C.M., Hong J.H. (2012). Irradiation promotes an M2 macrophage phenotype in tumor hypoxia. Front. Oncol..

[22] Crittenden M.R., Cottam B., Savage T., Nguyen C., Newell P., Gough M.J. (2012). Expression of NF-κB p50 in tumor stroma limits the control of tumors by radiation therapy. PLoS ONE.

[23] Vanpouille-Box C., Diamond J.M., Pilones K.A., Zavadil J., Babb J.S., Formenti S.C., Barcellos-Hoff M.H., Demaria S. (2015). TGFβ is a master regulator of radiation therapy-induced antitumor immunity. Cancer Res..

[24] Park S.S., Dong H., Liu X., Harrington S.M., Krco C.J., Grams M.P., Mansfield A.S., Furutani K.M., Olivier K.R., Kwon E.D. (2015). PD-1 restrains radiotherapy-induced abscopal effect. Cancer Immunol. Res..

[25] Keir M.E., Butte M.J., Freeman G.J., Sharpe A.H. (2008). PD-1 and its ligands in tolerance and immunity. Annu. Rev. Immunol..

[26] Ganss R., Hanahan D. (1998). Tumor microenvironment can restrict the effectiveness of activated antitumor lymphocytes. Cancer Res..

[27] Sznol M., Chen L. (2013). Antagonist antibodies to PD-1 and B7-H1 (PD-L1) in the treatment of advanced human cancer. Clin. Cancer Res..

[28] Maekawa N., Konnai S., Ikebuchi R., Okagawa T., Adachi M., Takagi S., Kagawa Y., Nakajima C., Suzuki Y., Murata S. (2014). Expression of PD-L1 on canine tumor cells and enhancement of IFN-γ production from tumor-infiltrating cells by PD-L1 blockade. PLoS ONE.

[29] Hartley G., Faulhaber E., Caldwell A., Coy J., Kurihara J., Guth A., Regan D., Dow S. (2017). Immune regulation of canine tumour and macrophage PD-L1 expression. Vet. Comp. Oncol..

[30] Shosu K., Sakurai M., Inoue K., Nakagawa T., Sakai H., Morimoto M., Okuda M., Noguchi S., Mizuno T. (2016). Programmed cell death ligand 1 expression in canine cancer. In Vivo.

[31] Maekawa N., Konnai S., Okagawa T., Nishimori A., Ikebuchi R., Izumi Y., Takagi S., Kagawa Y., Nakajima C., Suzuki Y. (2016). Immunohistochemical analysis of PD-L1 expression in canine malignant cancers and PD-1 expression on lymphocytes in canine oral melanoma. PLoS ONE.

[32] Kumar S.R., Kim D.Y., Henry C.J., Bryan J.N., Robinson K.L., Eaton A.M. (2017). Programmed death ligand 1 is expressed in canine B cell lymphoma and downregulated by MEK inhibitors. Vet. Comp. Oncol..

[33] Maekawa N., Konnai S., Takagi S., Kagawa Y., Okagawa T., Nishimori A., Ikebuchi R., Izumi Y., Deguchi T., Nakajima C. (2017). A canine chimeric monoclonal antibody targeting PD-L1 and its clinical efficacy in canine oral malignant melanoma or undifferentiated sarcoma. Sci. Rep..

[34] Hartley G., Elmslie R., Dow S., Guth A. (2018). Checkpoint molecule expression by B and T cell lymphomas in dogs. Vet. Comp. Oncol..

[35] Maekawa N., Konnai S., Nishimura M., Kagawa Y., Takagi S., Hosoya K., Ohta H., Kim S., Okagawa T., Izumi Y. (2021). PD-L1 immunohistochemistry for canine cancers and clinical benefit of anti-PD-L1 antibody in dogs with pulmonary metastatic oral malignant melanoma. npj Precis. Oncol..

[36] Gulay K.C.M., Aoshima K., Maekawa N., Suzuki T., Konnai S., Kobayashi A., Kimura T. (2022). Hemangiosarcoma cells induce M2 polarization and PD-L1 expression in macrophages. Sci. Rep..

[37] Brahmer J.R., Tykodi S.S., Chow L.Q., Hwu W.J., Topalian S.L., Hwu P., Drake C.G., Camacho L.H., Kauh J., Odunsi K. (2012). Safety and activity of anti-PD-L1 antibody in patients with advanced cancer. N. Engl. J. Med..

[38] Sato H., Okonogi N., Nakano T. (2020). Rationale of combination of anti-PD-1/PD-L1 antibody therapy and radiotherapy for cancer treatment. Int. J. Clin. Oncol..

[39] Takahashi Y., Yasui T., Tamari K., Minami K., Otani K., Isohashi F., Seo Y., Kambe R., Koizumi M., Ogawa K. (2017). Radiation enhanced the local and distant anti-tumor efficacy in dual immune checkpoint blockade therapy in osteosarcoma. PLoS ONE.

[40] Shaverdian N., Lisberg A.E., Bornazyan K., Veruttipong D., Goldman J.W., Formenti S.C., Garon E.B., Lee P. (2017). Previous radiotherapy and the clinical activity and toxicity of pembrolizumab in the treatment of non-small-cell lung cancer: A secondary analysis of the KEYNOTE-001 phase 1 trial. Lancet Oncol..

[41] Veterinary Cooperative Oncology Group (VCOG) (2016). Veterinary cooperative oncology group—Common terminology criteria for adverse events (VCOG-CTCAE) following chemotherapy or biological antineoplastic therapy in dogs and cats v1.1. Vet. Comp. Oncol..

[42] Nguyen S.M., Thamm D.H., Vail D.M., London C.A. (2015). Response evaluation criteria for solid tumours in dogs (v1.0): A Veterinary Cooperative Oncology Group (VCOG) consensus document. Vet. Comp. Oncol..

[43] Kanda Y. (2013). Investigation of the freely available easy-to-use software ‘EZR’ for medical statistics. Bone Marrow Transplant..

[44] Grimaldi A.M., Simeone E., Giannarelli D., Muto P., Falivene S., Borzillo V., Giugliano F.M., Sandomenico F., Petrillo A., Curvietto M. (2014). Abscopal effects of radiotherapy on advanced melanoma patients who progressed after ipilimumab immunotherapy. Oncoimmunology.

[45] Roger A., Finet A., Boru B., Beauchet A., Mazeron J.J., Otzmeguine Y., Blom A., Longvert C., de Maleissye M.F., Fort M. (2018). Efficacy of combined hypo-fractionated radiotherapy and anti-PD-1 monotherapy in difficult-to-treat advanced melanoma patients. Oncoimmunology.

[46] Yamaguchi O., Kaira K., Hashimoto K., Mouri A., Miura Y., Shiono A., Nishihara F., Murayama Y., Noda S.E., Kato S. (2019). Radiotherapy is an independent prognostic marker of favorable prognosis in non-small cell lung cancer patients after treatment with the immune checkpoint inhibitor, nivolumab. Thorac. Cancer.

[47] Antonia S.J., Villegas A., Daniel D., Vicente D., Murakami S., Hui R., Kurata T., Chiappori A., Lee K.H., de Wit M. (2018). Overall survival with durvalumab after chemoradiotherapy in stage III NSCLC. N. Engl. J. Med..

[48] Kong Y., Ma Y., Zhao X., Pan J., Xu Z., Zhang L. (2021). Optimizing the treatment schedule of radiotherapy combined with anti-PD-1/PD-L1 immunotherapy in metastatic cancers. Front. Oncol..

[49] Dovedi S.J., Adlard A.L., Lipowska-Bhalla G., McKenna C., Jones S., Cheadle E.J., Stratford I.J., Poon E., Morrow M., Stewart R. (2014). Acquired resistance to fractionated radiotherapy can be overcome by concurrent PD-L1 blockade. Cancer Res..

[50] Liniker E., Menzies A.M., Kong B.Y., Cooper A., Ramanujam S., Lo S., Kefford R.F., Fogarty G.B., Guminski A., Wang T.W. (2016). Activity and safety of radiotherapy with anti-PD-1 drug therapy in patients with metastatic melanoma. Oncoimmunology.

[51] Gray J.E., Villegas A., Daniel D., Vicente D., Murakami S., Hui R., Kurata T., Chiappori A., Lee K.H., Cho B.C. (2020). Three-year overall survival with durvalumab after chemoradiotherapy in Stage III NSCLC-update from PACIFIC. J. Thorac. Oncol..

[52] Wei J., Montalvo-Ortiz W., Yu L., Krasco A., Ebstein S., Cortez C., Lowy I., Murphy A.J., Sleeman M.A., Skokos D. (2021). Sequence of αPD-1 relative to local tumor irradiation determines the induction of abscopal antitumor immune responses. Sci. Immunol..

