# Supplementary material for: Enhanced Systemic Antitumour Immunity by Hypofractionated Radiotherapy and Anti-PD-L1 Therapy in Dogs with Pulmonary Metastatic Oral Malignant Melanoma
Source: Cancers (Basel). 2023 Jun 1;15(11):3013. doi: 10.3390/cancers15113013 (PMC10252299; doi:10.3390/cancers15113013)
Supplement: Supplementary file 1 [file cancers-15-03013-s001.zip › cancers-2361873-supplementary.pdf]

Supplementary Information for

Enhanced systemic antitumor immunity by hypofractionated radiotherapy and anti-PD-L1 therapy in dogs with pulmonary metastatic oral malignant melanoma

Tatsuya Deguchi†, Naoya Maekawa†, Satoru Konnai*, Ryo Owaki, Kenji Hosoya, Keitaro Morishita, Motoji Nakamura, Tomohiro Okagawa, Hiroto Takeuchi, Satoshi Takagi, Sangho Kim, Ryohei Kinoshita, Yurika Tachibana, Madoka Yokokawa, Yukinari Kato, Yasuhiko Suzuki, Shiro Murata, Kazuhiko Ohashi

†These authors contributed to this work equally.

*Corresponding author
email: konnai@vetmed.hokudai.ac.jp

Table S1. Baseline characteristics of c4G12-treated dogs.

Dog #	Breed	Sex	Age (Year)	Body weight (kg)	Site of primary tumor	PD-L1 expression	Prior therapy	Concomitant therapy
1	Pug	Male, castrated	11	8.4	Mandible	+	Surgery	None
2	Miniature dachshund	Male, castrated	14	8.8	Maxilla	+	Radiation	None
3	Golden retriever	Male, castrated	10	26.4	Maxilla	+	Surgery	None
4	Beagle	Female, spayed	11	9.7	Maxilla	+	Surgery	Surgery
5	American cocker spaniel	Male, castrated	13	8.9	Mandible	+	Surgery	None
6	Yorkshire terrier	Male	14	2.0	Mandible	+	Surgery	None
7	Kaninchen dachshund	Female, spayed	13	5.7	Mandible	+	Surgery, chemotherapy	None
8	Miniature dachshund	Male	13	6.5	Mandible	+	Surgery	None
9	Mix	Female	12	16.8	Maxilla	-	Surgery	None
10	Miniature dachshund	Male	13	4.7	Unspecified	+	Surgery, radiation	None
11	Miniature dachshund	Female	15	4.4	Mandible	+	Surgery	None
12	Toy poodle	Female, spayed	13	4.6	Maxilla	+	Radiation	None
13	Miniature dachshund	Male	14	5.2	Maxilla	ND	Radiation	None
14	Chihuahua	Female, spayed	13	2.3	Mandible	+	Surgery	None
15	Pug	Female, spayed	14	7.6	Mandible	+	Surgery	None
16	Papillon	Male, castrated	14	3.7	Unspecified	+	Surgery	None
17	Flat-coated retriever	Female, spayed	8	26.7	Maxilla	+	Surgery	None
18	Miniature dachshund	Male, castrated	14	8.3	Mandible	+	None	None
19	Mix	Female, spayed	15	9.0	Maxilla	+	Surgery	None
20	Labrador retriever	Female, spayed	12	29.0	Mandible	+	Surgery	None
21	Miniature dachshund	Male, castrated	14	5.7	Maxilla	+	Radiation	None
22	Pomeranian	Male	12	2.6	Maxilla	+	Surgery, radiation	None
23	Pekingese	Male, castrated	8	6.5	Maxilla	+	Radiation	None
24	Mix	Male, castrated	11	15.4	Maxilla	+	Radiation	None
25	Labrador retriever	Female	11	22.5	Mandible	+	Radiation	None
26	American cocker spaniel	Male	10	8.0	Maxilla	+	Radiation	None

27	Miniature dachshund	Female, spayed	15	4.2	Mandible	-	Surgery, radiation	Surgery
28	Golden retriever	Male, castrated	14	29.0	Maxilla	+	Surgery, radiation	Surgery
29	Golden retriever	Male, castrated	11	26.0	Maxilla	+	Surgery, radiation	None
30	Miniature dachshund	Female, spayed	10	4.8	Maxilla	+	None	Radiation
31	Miniature dachshund	Male, castrated	15	4.7	Mandible	+	Surgery	Radiation
32	Pug	Female	14	6.0	Mandible	+	None	Radiation
33	Miniature dachshund	Female, spayed	15	4.2	Mandible	ND	None	Radiation
34	Miniature dachshund	Male, castrated	14	4.6	Mandible	+	None	Radiation
35	Toy poodle	Female	13	3.7	Maxilla	+	None	Radiation
36	Mix	Female, spayed	14	6.6	Maxilla	+	None	Radiation
37	Chihuahua	Male, castrated	13	4.7	Maxilla	ND	None	Radiation
38	Toy poodle	Female, spayed	14	5.5	Maxilla	+	None	Radiation, surgery
39	Mix	Male, castrated	13	3.6	Mandible	+	None	Radiation

ND, not determined.

Table S2. Summary of c4G12 treatment.

Dog #	c4G12 treatment duration	No. of c4G12 doses	Amount of dosage	Timing of RT	Overall survival (days)	Intrathoracic response	Cause of death
1	91	7	Weeks 0–8: 2 mg/kg, then 5 mg/kg	No RT	91*	PD	NA (Censored)
2	42	3	2 mg/kg	No RT	81	PD	Local
3	36	3	2 mg/kg	No RT	36*	PD	NA (Censored)
4	335	25	Weeks 0–12: 5 mg/kg, then 2 mg/kg	No RT	362	CR	Metastasis
5	98	7	5 mg/kg	No RT	108	PD	Local
6	44	4	5 mg/kg	No RT	44	PD	Metastasis
7	35	2	5 mg/kg	No RT	35*	NE	NA (Censored)
8	42	3	5 mg/kg	No RT	44*	PD	NA (Censored)
9	128	9	5 mg/kg	No RT	128*	PD	NA (Censored)
10	28	3	5 mg/kg	No RT	28*	NE	NA (Censored)
11	28	2	5 mg/kg	No RT	44	PD	Metastasis
12	35	3	5 mg/kg	No RT	58	PD	Metastasis
13	118	8	5 mg/kg	No RT	118	PD	Metastasis
14	185	13	5 mg/kg	No RT	185	PD	Metastasis
15	26	2	5 mg/kg	No RT	40	PD	Metastasis
16	15	1	5 mg/kg	No RT	42*	PD	NA (Censored)
17	138	10	2 mg/kg	No RT	138	PD	Metastasis
18	42	3	5 mg/kg	No RT	53	PD	Metastasis
19	152	11	5 mg/kg	No RT	152	SD	Metastasis
20	14	2	2 mg/kg	No RT	63	NE	Metastasis
21	82	6	5 mg/kg	Previous RT	88	PD	Metastasis
22	168	11	5 mg/kg	Previous RT	168	CR	Chronic kidney disease
23	271	19	5 mg/kg	Previous RT	271*	CR	NA (Censored)
24	61	5	5 mg/kg	Previous RT	61	PD	Metastasis
25	98	6	2 mg/kg	Previous RT	98	SD/PR	Local
26	101	7	5 mg/kg	Previous RT	101*	PD	NA (Censored)

27	750	54	5 mg/kg	Previous RT	750	CR	Metastasis
28	399	28	Weeks 0–42: 2 mg/kg, then 5 mg/kg	Previous RT	399	CR	Metastasis
29	124	9	2 mg/kg	Previous RT	124*	PD	Alive (Censored)
30	2	1	5 mg/kg	Concurrent RT	2	NE	Metastasis
31	89	6	5 mg/kg	Concurrent RT	93	PD	Metastasis
32	188	13	5 mg/kg	Concurrent RT	194	SD/PR	Local
33	28	3	5 mg/kg	Concurrent RT	28*	PD	NA (Censored)
34	56	4	5 mg/kg	Concurrent RT	56*	PD	NA (Censored)
35	129	10	5 mg/kg	Concurrent RT	129	PD	Metastasis
36	116	9	5 mg/kg	Concurrent RT	116*	PD	NA (Censored)
37	175	11	5 mg/kg	Concurrent RT	191	PD	Metastasis
38	131	8	5 mg/kg	Concurrent RT	131*	SD/PR	Alive (Censored)
39	20	2	5 mg/kg	Concurrent RT	101	NE	Metastasis

Overall survival (days) of dogs was defined as time from the first c4G12 dose to death.

SD/PR represents non-CR/non-PD in dogs with non-measurable pulmonary lesions at baseline.

*Censored due to loss to follow-up or alive at the end of the study period.

CR, complete response; PR, partial response; SD, stable disease; PD, progressive disease; NE, not evaluable; NA, not applicable.

Table S3. Summary of dogs in RT-only group.

Breed	Sex	Age (Year)	Overall survival from RT (days)
Golden retriever	Female, spayed	14	13*
Miniature dachshund	Male, castrated	14	48
Miniature dachshund	Female, spayed	13	14
Miniature dachshund	Female, spayed	14	48
Mix	Male, castrated	13	21
Chihuahua	Male, castrated	13	152
Miniature dachshund	Male	13	79
Miniature dachshund	Male	16	20*
Pug	Female, spayed	14	14*
Miniature schnauzer	Male	12	21*

Overall survival (days) of dogs was defined as time from the first RT dose to death.

*Censored due to loss to follow-up.

Table S4. Adverse events related to RT.

	RT-only (*n* =10)		Previous RT (*n* = 9)		Concurrent RT (*n* = 10)	
	Any grade	Grade 3	Any grade	Grade 3	Any grade	Grade 3
Any	2 (20)	0	3 (33)	0	6 (60)	1 (10)
Alopecia	1 (10)	0	1 (11)	0	2 (20)	0
Pruritus	1 (10)	0	1 (11)	0	2 (20)	0
Mucositis/stomatitis	0	0	1 (11)	0	1 (10)	0
Skin ulceration	0	0	2 (22)	0	0	0
AST	NE	NE	NE	NE	2 (20)	1 (10)

No. of dogs with event (%) are shown.

Adverse events considered to be related to RT by the investigators are listed.

AST, aspartate aminotransferase; NE, not evaluated.

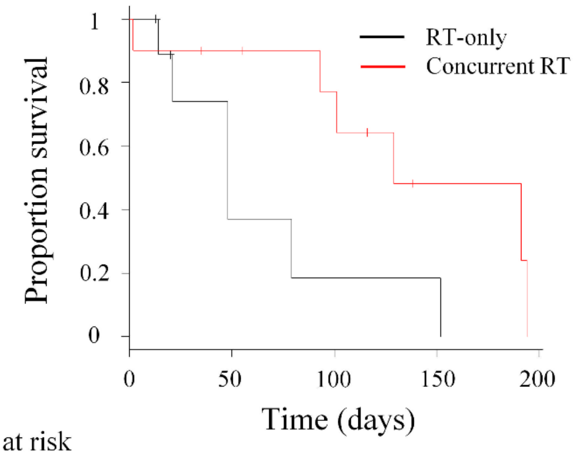

Number at risk						
RT-Only	10	2	1	1	0	$P = 0.036$
Concurrent RT	10	8	6	2	0	

Figure S1. OS of dogs treated with RT only or c4G12 immunotherapy plus concurrent RT. Kaplan-Meier curves for OS from the initiation of RT are shown. Marks on the line indicate censored data. Statistical analysis was performed using the log-rank test.
